# Analysis of PD-L1 promoter methylation combined with immunogenic context in pancreatic ductal adenocarcinoma

**DOI:** 10.1007/s00262-024-03745-y

**Published:** 2024-06-04

**Authors:** Xinyuan Chen, Shuangni Yu, Jie Chen, Xianlong Chen

**Affiliations:** 1 Department of Pathology, Peking Union Medical College Hospital, Chinese Academy of Medical Sciences & Peking Union Medical College, Beijing, 100730 China; 2 4+4 Medical Doctor Program, Chinese Academy of Medical Sciences & Peking Union Medical College, Beijing, China

**Keywords:** Pancreatic ductal adenocarcinoma, Methylation, PD-L1, PD-L2, Survival, Epigenetic biomarker

## Abstract

Despite the successful application of programmed cell death ligand 1 (PD-L1)-blocking strategies in some types of cancers and well-established prognostic indicators in pancreatic ductal adenocarcinoma (PDAC), the biological and clinical implications of the methylation status of PD-L1/PD-L2 in PDAC remain largely unknown. Therefore, this study aimed to explore the biological role of PD-L1/PD-L2 methylation and its association with clinicopathological features, clinical outcomes, and the immune microenvironment by analyzing the data on PD-L1/PD-L2 methylation and mRNA expression in PDAC cohorts obtained from the Cancer Genome Atlas and International Cancer Genome Consortium. The correlation between PD-L1 promoter methylation and PD-L1 expression and survival was further validated in an independent validation cohort (Peking Union Medical College Hospital [PUMCH] cohort) using pyrosequencing and immunohistochemistry. These results demonstrated that hypomethylation of the PD-L1 promoter was strongly associated with upregulated PD-L1 expression and shorter overall survival in PDAC. Multivariate Cox regression analyses revealed that the PD-L1 promoter methylation was an independent prognostic factor. PD-L1 promoter hypomethylation and high expression were related to aggressive clinical phenotypes. Moreover, both PD-L1 and PD-L2 methylation correlated with immune cell infiltration and the expression of immune checkpoint genes. PD-L1 promoter methylation status was further validated as an independent prognostic biomarker in patients with PDAC using the PUMCH cohort. The prognostic significance of PD-L1 promoter methylation was more discriminative in tumors with perineural/lymphovascular invasion and distant metastasis than in those without perineural/lymphovascular invasion and distant metastasis. In summary, the methylation status of the PD-L1 promoter is a promising biomarker for survival outcomes, immune infiltration, and the potential immune benefits of immunotherapy in PDAC.

**Supplementary Information:**

The online version contains supplementary material available at 10.1007/s00262-024-03745-y.

## Background

Pancreatic cancer ranks as the third leading cause of cancer-related mortality worldwide [1, 2]. Pancreatic ductal adenocarcinomas (PDACs), constituting approximately 90% of pancreatic cancers, represent an exceptionally deadly malignancy, boasting a mere 10% 5-year survival rate [3, 4]. In contrast to several cancers detectable in their early stages, PDAC typically manifests as advanced-stage neoplasms upon diagnosis for most patients. Apart from surgery, other therapeutic modalities, encompassing both adjuvant and neoadjuvant chemotherapy and radiotherapy, yield unsatisfactory outcomes, with marginal enhancements in the 5-year survival rate [5].

Immune checkpoint inhibitor (ICI) immunotherapy targeting programmed cell death 1 (PD-1) or its ligand, PD-1 ligand 1 (PD-L1), also exhibits restricted efficacy in advanced PDAC on account of its immunologically "cold" tumor profile, characterized by a low mutation burden and diminished expression of CD8, PD-1, and PD-L1 [6–8]. Notably, while PD-L1 (*CD274*) expression demonstrates restricted predictive capacity for ICI response in PDAC [9], heightened PD-L1 expression correlates with adverse prognosis in PDAC [10, 11].

In addition to PD-L1, PD-1 has another ligand, PD-L2 (*PDCD1LG2*), which is upregulated in some malignancies and immune cells, such as macrophages and dendritic cells (DCs). High expression of PD-L2 alters the tumor microenvironment (TME) by increasing PD-1^+^ tumor-infiltrating lymphocytes. These lymphocytes can provide prognostic assessment and evaluate the response to immunotherapies [12]. In PDAC, high PD-L2 expression has been associated with worse survival outcomes and improved predictive performance for survival outcomes when intratumoral CD3^+^ cells and stromal FOXP3^+^ cells are incorporated [13].

Notwithstanding the common clinical application of PD-L1/PD-L2 expression as a prognostic biomarker in various cancers, including PDAC, the regulatory mechanisms underlying PD-L1/PD-L2 expression remain unclear. Many studies have demonstrated that DNA hypermethylation at specific sites, especially in promoters, may induce the downregulation of corresponding gene expression, and vice versa [14]. Consequently, alterations in methylation can modify the cellular phenotype and reshape the TME, resulting in cell immortalization and immune invasion. DNA methylation can regulate PD-L1/PD-L2 expression in many malignancies, including melanoma, colorectal cancer, and other tumors [15, 16]; however, it has not been investigated in PDAC.

To date, few studies have systematically examined the role of PD-L1/PD-L2 methylation in the regulation of expression and immune cell infiltration, as well as its prognostic and therapeutic significance in PDAC. Therefore, this study aimed to explore the relationship between PD-L1/PD-L2 methylation and survival outcomes, gene expression, and alterations in the immune microenvironment using public datasets. Additionally, the study sought to validate these conclusions in a large retrospective cohort (the Peking Union Medical College Hospital [PUMCH] cohort) to clarify their regulatory mechanisms and prognostic potential.

## Methods

### Datasets acquisition and analysis

RNA sequencing data, methylation data, and associated clinicopathological information for PDAC were downloaded from The Cancer Genome Atlas (TCGA) and International Cancer Genome Consortium (ICGC) databases. Clinicopathological information from TCGA and ICGC is summarized in Table S1.

We utilized the R package “minfi” to preprocess and exploratorily analyze the methylation data obtained from Infinium HumanMethylation450 BeadChip arrays in TCGA and ICGC cohorts. Following preprocessing using ‘minfi,’ we computed beta values to denote the methylation levels of CpG sites, which were subsequently employed for correlation analysis and sample stratification. The β value was applied to represent the methylation status of probes, and is defined as the ratio of methylated intensity to total intensity. For transcriptomic analysis, fragments per kilobase per million mapped fragments were acquired from TCGA, while microarray data were obtained from the ICGC datasets.

### Molecular expression association and immune checkpoint genes (ICGs)

Hu et al. assembled a panel of 74 genes linked to an immune checkpoint [17]. Correlation coefficients between the methylation/expression profiles of PD-L1/PD-L2 and expression profiles of ICGs were computed to illustrate the potential mechanisms of PD-L1/PD-L2 methylation in signaling pathways related to immune checkpoint.

### Gene set variation analysis (GSVA) and immune cell infiltration estimation

To investigate disparities in biological processes among distinct methylation status subgroups, we conducted GSVA enrichment employing the R package 'GSVA.' Gene sets linked to tumor immune microenvironment-related biological processes and responses to ICI immunotherapy were utilized in the GSVA [18].

The R package “xCell” was utilized to calculate the relative abundance of each TME infiltrating immune cell using transcriptomic data. “xCell” is a robust method based on gene signatures obtained from single sample gene set enrichment analysis (ssGSEA) using transcriptomes. It harmonizes pure human cell-type transcriptomes and applies a curve fitting approach for linear comparison of cell types to deduce immune cell types in the TME [19].

### PUMCH cohort

To assess the clinical value of PD-L1/PD-L2 methylation, we recruited 291 patients with PDAC who underwent standard surgical procedures, ensuring statistical adequacy through prior sample size calculation. All cases were histologically confirmed at Peking Union Medical College Hospital (PUMCH), Beijing, China, between 2015 and 2019. Raw data from the PUMCH cohort were extracted from electronic medical records and pathology reports, summarized in Table S2. Survival outcome data were retrospectively gathered via telephone interviews. The intervals between surgery and cancer-specific demise or the last follow-up (October 2020) were computed as disease-specific death occurrences. This study received approval from the Ethical Review Committee of PUMCH (S-K1593). All procedures involving human participants and sample acquisition adhered to the principles outlined in the 1964 Helsinki Declaration or equivalent ethical standards.

### Immunohistochemistry (IHC) and PD-L1 expression evaluation

IHC analysis was conducted using primary antibodies targeting PD-L1. Immunohistochemical staining was carried out with an automated immunostainer, adhering strictly to the manufacturer’s recommended standard protocol. The tumor proportion score (TPS) is a well-established scoring method for assessing PD-L1 expression. It is calculated from the proportion of PD-L1 + tumor cells among all tumor cells [20]. In line with clinical practice and prior investigations, the predefined cutoff was set at 1% [21].

### DNA isolation and pyrosequencing

Genomic DNA was extracted from the PUMCH cohort following the manufacturer’s instructions. Pyrosequencing was conducted to determine the methylation status of cg19724470. Briefly, extracted DNA underwent bisulfite treatment to convert unmethylated cytosine to uracil. The bisulfite-converted DNA was immediately subjected to polymerase chain reaction (PCR) amplification to serve as a template for detecting DNA methylation, specifically targeting the cg19724470 CpG sites. The primers for amplifying cg19724470 were as follows: forward primer 5’- TTTGGATACGGGTTTAAGTTTATCG-3’ and reverse primer 5’- AAAATCCTAAACCTACTTAACGCA-3’.

### Statistical analysis

Statistical analyses were conducted using R version 4.2.2. In our study, we assessed the correlation between methylation status and clinicopathologic characteristics using either Pearson’s χ2 test or Fisher’s exact test, as appropriate. Correlations between continuous variables in our study were presented using the Spearman ρ coefficient. Kaplan–Meier survival analysis was performed to examine the association between the expression/methylation profiles of PD-L1/PD-L2 with overall survival (OS) in PDAC using the R package “Survminer”. The optimal cutoff values of Kaplan–Meier analysis were derived from minimum *p*-value approaches. Univariate and multivariate analyses were conducted using a Cox proportional hazards regression model to estimate the hazard ratios (HRs) and corresponding 95% confidence intervals (CIs). The statistical significance was set: *: *p* < 0.05; **: *p* < 0.01; ***: *p* < 0.001.

## Results

### Comprehensive characteristics of PD-L1/PD-L2 methylation in PDAC

To explore the methylation status of PD-L1/PD-L2 in PDAC, we identified two probes located in PD-L1 (cg19724470 and cg02823866) and four probes located in PD-L2 (cg14374994, cg14351952, cg14133064, and cg07211259) based on annotation with the Infinium HumanMethylation450 Bead-Chip. Among these six probes, cg19724470 and cg02823866 are situated in the promoter regions of PD-L1 (cg19724470: 5’UTR; cg02823866: TSS200). cg14351952 and cg07211259 are positioned in the 5’UTR and TSS200 of PD-L2, while the remaining two probes are located in the gene body of PD-L2. Detailed information on these probes is provided in Table S3. Then, a total of 173 patients from the TCGA cohort and 174 patients from the ICGC cohort were downloaded and included in our study to match expression, methylation, and clinicopathological data.

Next, using the beta values calculated by minimum *p*-value approaches of the methylation probes as the cut-off value, we categorized the TCGA and ICGC cohorts into hypermethylated and hypomethylated subgroups for each probe. We compared the clinicopathological characteristics of the patients in the subgroups for each probe (Tables S4 and S5). No significant differences were observed for most factors in each subgroup. Notably, the ICGC data showed that the histological grades were significantly more malignant in the hypomethylated subgroup of cg07211259 (*p* = 0.002; TCGA: *p* = 0.555). Additionally, sex differences were observed between the cg19724470 subgroups in the ICGC cohort (*p* = 0.034; TCGA: *p* = 0.759).

### Expression-methylation correlation of PD-L1/PD-L2 in patients with PDAC

**Fig. 1:**
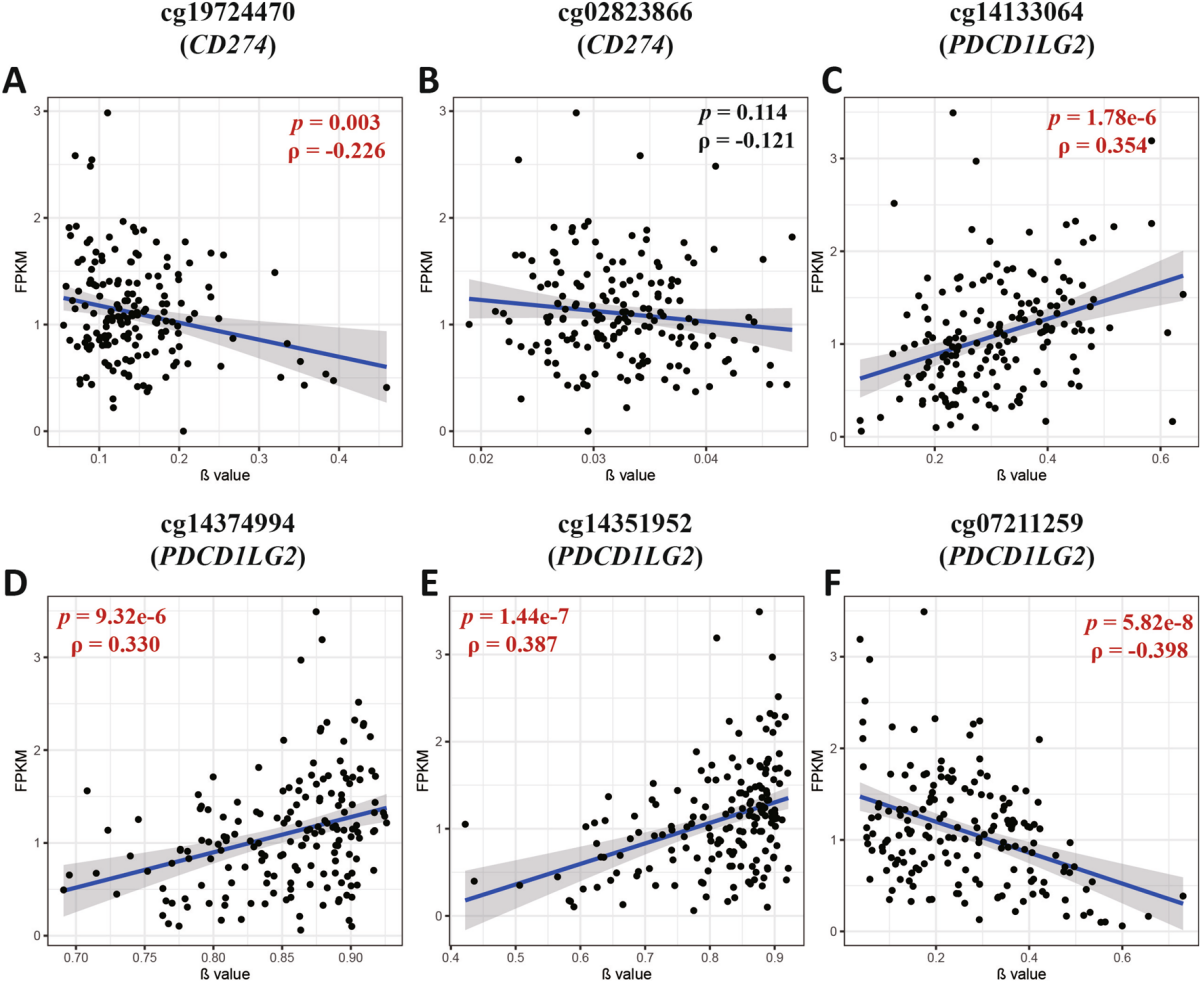
PD-L1/PD-L2 methylation status was correlated with corresponding *CD274/PDCD1LG2* expression in PDAC. **A**–**B**. The correlation of cg19724470 and cg02823866 with *CD274* expression in the TCGA cohort; **C**–**E**. The correlation of cg14133064, cg14374994, cg14351952, and cg07211259 with *PDCD1LG2* expression in the TCGA cohort. *p* values < 0.05 were marked in red

PD-L1/PD-L2 have been reported to play oncogenic roles that are independent of immune checkpoint regulation in multiple malignancies. To investigate the role of these CpG methylation loci in regulating *CD274/PDCD1LG2* expression, we performed a correlation analysis between these probes and the corresponding gene expression in the TCGA cohort. The cg19724470 (*CD274*) and cg07211259 (*PDCD1LG2*) CpG loci exhibited a significant negative linear correlation with the expression of the corresponding genes, suggesting that these two CpG sites are likely the primary epigenetic elements regulating the expression of *CD274/PDCD1LG2* in patients with PDAC (Fig. 1). However, cg14133064, cg14374994, and cg14351952 exhibited a significant positive linear correlation with the expression of *PDCD1LG2*, indicating that a more complex modification pattern may exist in the regulation of *PDCD1LG2* expression.

### Survival outcomes prediction with PD-L1/PD-L2 methylation status in patients with PDAC

**Fig. 2:**
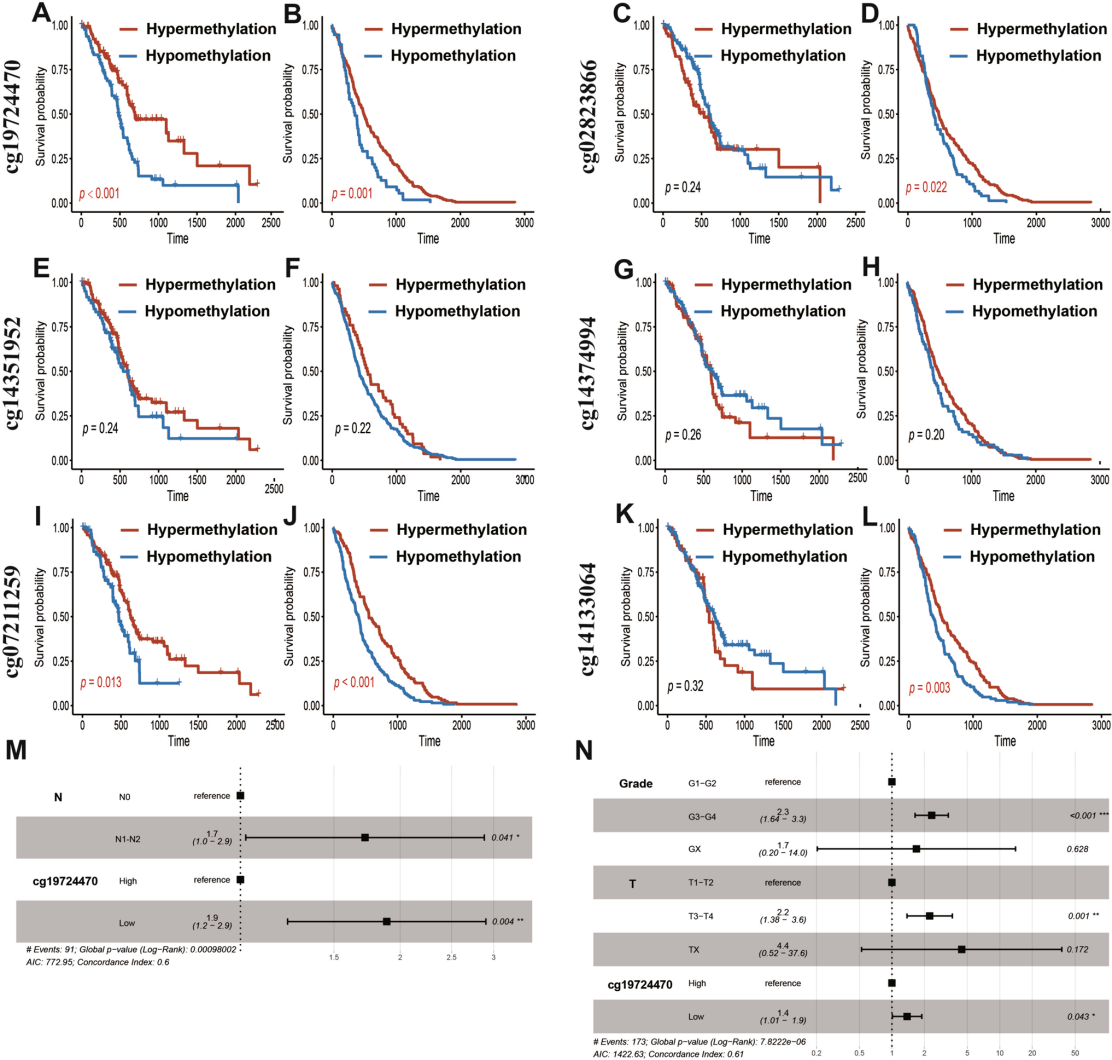
PD-L1 promoter methylation (cg19724470) predicted overall survival in PDAC. **A**–**L**. Kaplan–Meier survival analysis of cg19724470, cg02823866, cg14351952, cg14374994, cg07211259, and cg14133064 for overall survival of patients from the TCGA and ICGC cohorts, respectively. The best cutoff was applied in each Kaplan–Meier analysis. The blue line represents the hypomethylation group, and the red lines represent the hypermethylation group. **M**–**N**. Multivariate Cox analysis of cg19724470 methylation and the clinicopathological factors significant in the univariate Cox analysis (*p* < 0.05) from the TCGA and ICGC cohorts, respectively. *p* values < 0.05 were marked in red

To unveil the prognostic significance of the methylation status of PD-L1/PD-L2 in PDAC, we initially conducted a univariate Cox proportional hazards analysis encompassing these six probes and clinicopathological features across both the TCGA and ICGC cohorts (Table S6 and S7). Regarding methylation probes, the results revealed that the hypomethylation of cg19724470 was associated solely with poor survival outcomes in the TCGA cohort (TCGA: HR 1.92, *p* = 0.003; ICGC: HR 1.14, *p* = 0.405), whereas hypomethylation of cg07211259 indicated an unfavorable OS solely in the ICGC cohort (TCGA: HR 1.40, *p* = 0.121; ICGC: HR 1.62,* p* = 0.002). Kaplan–Meier survival analysis corroborated that only hypomethylation of cg19724470 exhibited a strong association with poor OS across both the TCGA and ICGC cohorts utilizing optimal cutoff values (ICGC:* p* = 0.001; TCGA:* p* < 0.001; Fig. 2A–L).

Regarding clinicopathological information, only N stage emerged as a significant predictor of poor survival outcomes in the TCGA cohort (N0 vs. N1 [*p* = 0.032]), while histological grade (G1/G2 vs. G3/G4 [*p* < 0.001]) and T stage (T1/T2 vs. T3/T4 [*p* = 0.038]) were significant predictors of poor OS in the ICGC cohort. Furthermore, through the amalgamation of methylation probes and statistically significant clinicopathological features (*p* < 0.05), cg19724470 was identified as the sole independent risk factor for predicting poor survival outcomes via multivariate Cox analysis in both TCGA and ICGC cohorts (Fig. 2M, N and Figure S1). In summary, our results validate that the cg19724470 methylation status independently and robustly contributes to the survival outcomes of PDAC.

### Relevance of the expression/methylation profiles of PD-L1/PD-L2 with immune cell infiltration in the TME

**Fig. 3:**
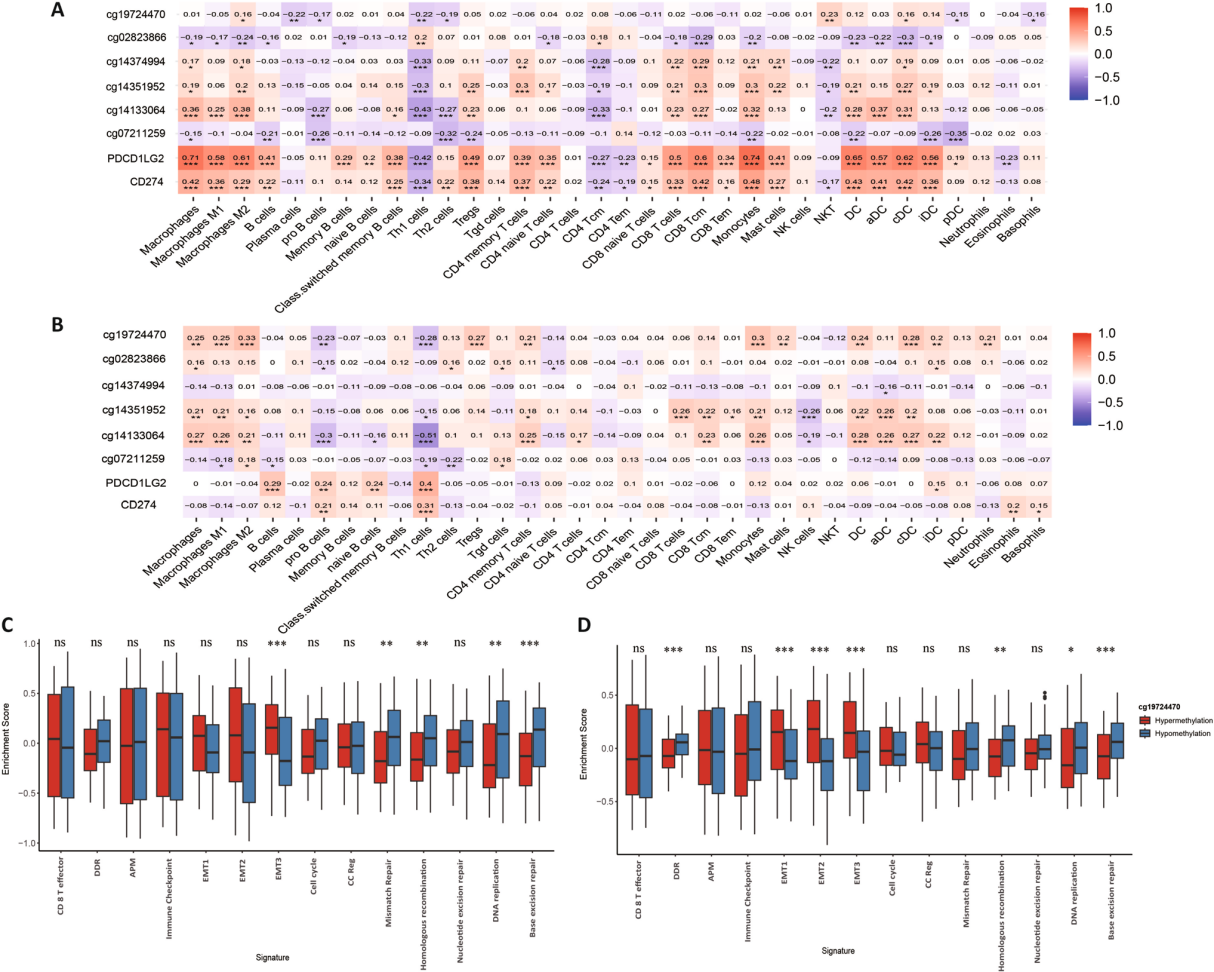
PD-L1/PD-L2 expression and corresponding methylation probes were correlated with immune cell infiltration and immunotherapy response in PDAC. **A**–**B**. The correlation heatmap of PD-L1/PD-L2 expression and related methylation probes with 34 types of immune cells in the TCGA and ICGC cohorts, respectively. The correlation coefficients and statistical significance are shown in each grid; **C**–**D**. Relative enrichment scores of 14 immunotherapy response-related pathways in hypermethylation and hypomethylation subgroups of cg19724470 in the TCGA and ICGC cohorts, respectively. *:* p* < 0.05; **: *p* < 0.01; ***: *p* < 0.001; ns, not significant

The expression of *CD274/PDCD1LG2* is pivotal in immune cell infiltration in the TME across various cancers. Thus, we hypothesized that the expression/methylation profiles of PD-L1/PD-L2 might correlate with immune cell infiltration in the TME. To investigate this hypothesis, we correlated 34 immune cell types, as determined by xCell, with the expression/methylation levels of PD-L1/PD-L2 using both TCGA and the ICGC cohorts. In the TCGA database, we observed a more significant correlation between the methylation status of these loci and the infiltration of macrophages, DCs, and T cells, which aligns with findings from the ICGC cohort. Notably, we found significant negative correlations between cg19724470 and Th1 cells in both the TCGA and ICGC cohorts (TCGA:ρ = − 0.22; ICGC: ρ = − 0.28; Fig. 3A, B).

Furthermore, to explore the role of cg19724470 in response to anti-PD-L1 immunotherapy for PDAC, we performed a correlation analysis between cg19724470 subgroups and 14 core biological pathways in TCGA and ICGC cohorts. We found that hypermethylation of cg19724470 was associated with a higher epithelial-mesenchymal transition (EMT) score (TCGA: *p* < 0.001; ICGC:* p* < 0.001), while a higher homologous recombination (TCGA: *p* = 0.009; ICGC: *p* = 0.003), DNA replication (TCGA: *p* < 0.001; ICGC: *p* = 0.030), and base excision repair (TCGA: *p* < 0.001; ICGC:* p* < 0.001) scores were observed in the hypomethylated subgroup. These results indicate that the methylation status of cg19724470 may induce different immune escape patterns, potentially leading to different responses to ICI immunotherapy (Fig. 3C–D).

### Association of the expression/methylation profiles of PD-L1/PD-L2 with ICGs expression

**Fig. 4:**
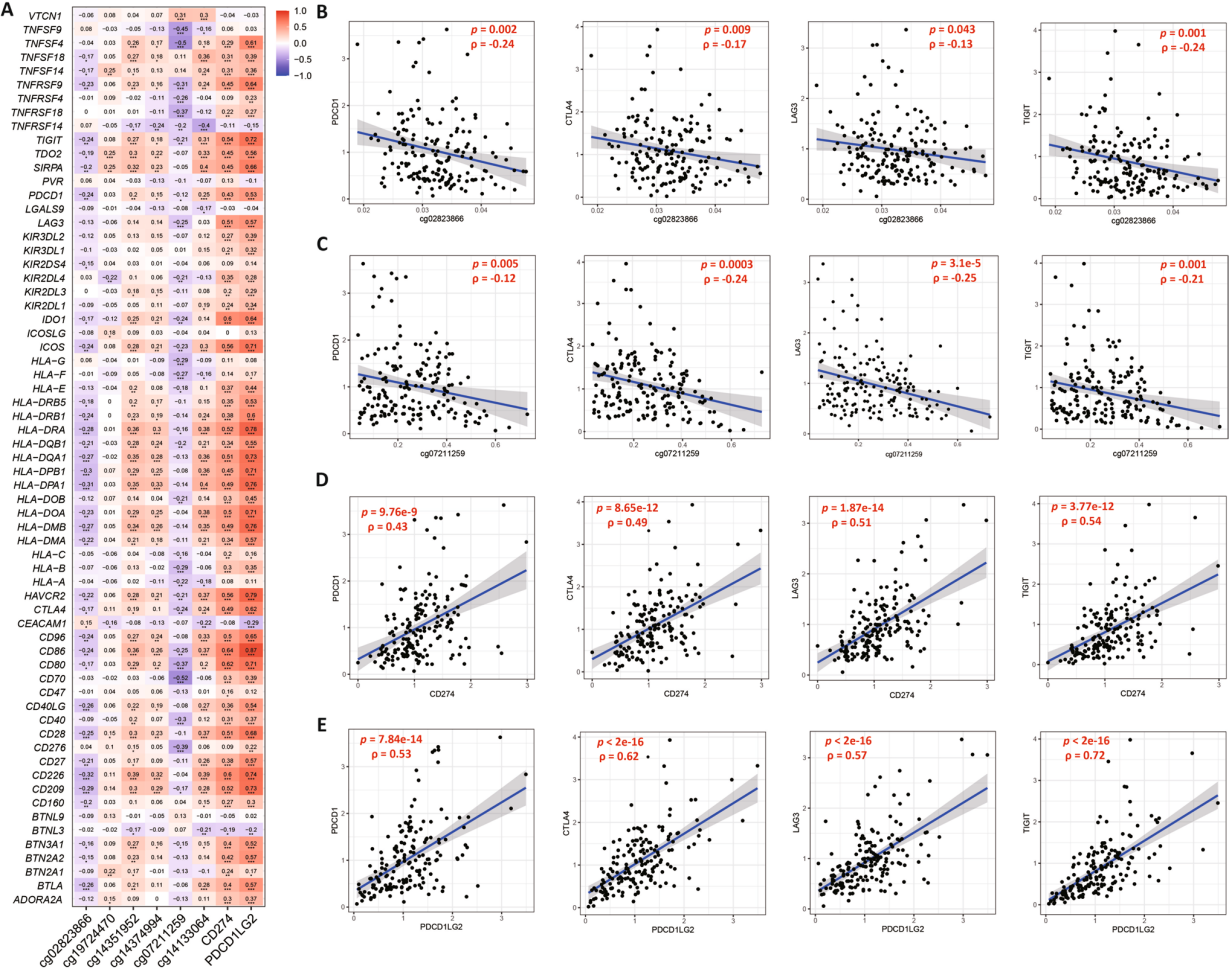
*CD274/PDCD1LG2* expression and corresponding methylation probes were correlated with the expression of ICGs in PDAC. **A**. The correlation heatmap of PD-L1/PD-L2 expression and related methylated CpG sites with 65 detected ICGs in the TCGA cohort; **B**–**E**. The correlation of cg02823866 or cg07211259, and *CD274/PDCD1LG2* expression with immune checkpoint molecules *PDCD1*, *CTLA4*, *LAG3*, and *TIGIT*, respectively, in the TCGA cohort. *p* values < 0.05 were marked in red

ICGs are involved in immune costimulatory and inhibitory pathways, which play critical roles in avoiding self-reactivity. In this study, we analyzed the association between *CD274*/*PDCD1LG2* expression and the corresponding methylation of 65 ICGs. We found that upregulated expression of *CD274/PDCD1LG2* and hypomethylation of cg07211259/cg02823866 were significantly and positively correlated with the expression of most ICGs in the TCGA cohort (Fig. 4A). Specifically, to assess the therapeutic potential of other ICIs against PD-L1/PD-L2 inhibitors, we analyzed the relationship of *CD274/PDCD1LG2* expression and cg07211259/cg02823866 methylation with other therapeutic immune checkpoint molecules (*PDCD1*, *CTLA4, LAG3*, and *TIGIT*). We found that *CD274/PDCD1LG2* expression was significantly and positively correlated with the expression of these genes in the TCGA cohort. Consistent with the biological regulatory mechanism of methylation, a significant negative linear correlation was observed between cg07211259/cg02823866 methylation and *PDCD1*, *CTLA4*, *LAG3*, and *TIGIT* (Fig. 4B–E). However, cg19724470 did not significantly correlate with these molecules (Figure S2). We confirmed our results using an ICGC cohort (Figure S3). Our findings highlight the potential clinical benefits of co-targeted immunotherapy for pancreatic cancer.

### Validation of prognostic significance of PD-L1 expression and methylation in the PUMCH cohort

Based on prior findings from the TCGA and ICGC cohorts, we identified the CpG site cg19724470 via pyrosequencing and verified PD-L1 expression by IHC in our PUMCH cohort. Employing a 50% cutoff generated via minimum *p*-value approaches, we stratified all patients into hypermethylated and hypomethylated subgroups, while further categorizing the two subgroups based on PD-L1 TPS, with a cutoff of 1%. Initially, we compared the clinicopathological characteristics across different subgroups. Our data revealed that histological grade and N stage showed a significant increase in malignancy within the hypomethylated subgroup (Table S8).

**Fig. 5:**
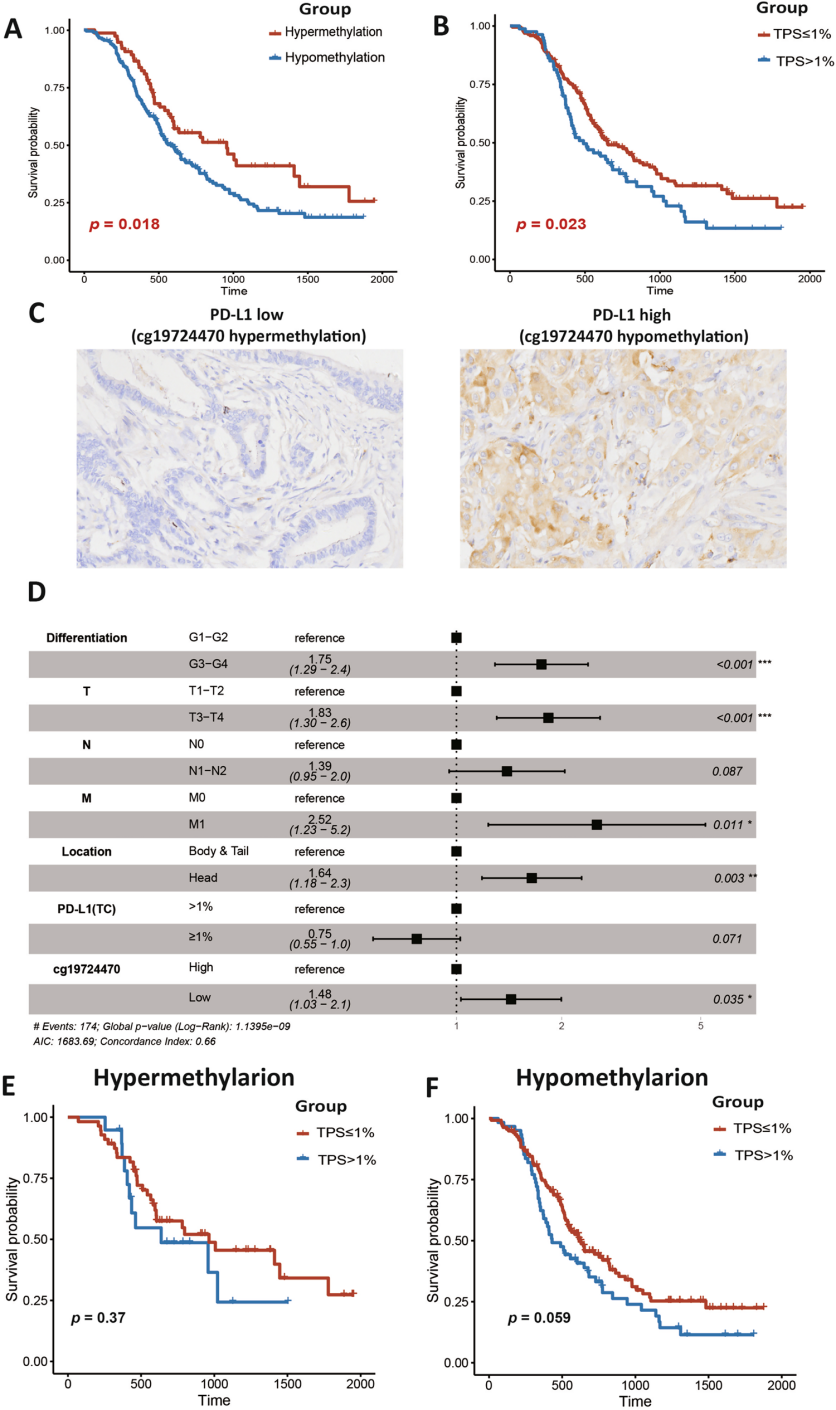
cg19724470 methylation and PD-L1 expression were associated with overall survival in PUMCH cohort. **A**. Kaplan–Meier survival analysis of cg19724470 methylation; **B**. Kaplan–Meier survival analysis of PD-L1 TPS; **C**. The representative IHC slides of PD-L1 in the hypermethylation and hypomethylation subgroups of PUMCH cohort; **D**. Multivariate Cox analysis of cg19724470 methylation, PD-L1 TPS, and the clinicopathological factors significant in the univariate Cox analysis (*p* < 0.05); **E**. Kaplan–Meier survival analysis of PD-L1 TPS in the hypermethylation subgroup; **F**. Kaplan–Meier survival analysis of PD-L1 TPS in the hypomethylation subgroup. PD-L1, programmed cell death ligand 1; TC, tumor cell. *p* values < 0.05 were marked in red

Consistent with findings from the TCGA and ICGC cohorts, hypomethylation of cg19724470 was significantly related to poor survival outcomes (*p* = 0.018; Fig. 5A) in the PUMCH cohort. Subsequently, to elucidate the prognostic significance of expression levels of PD-L1 in our cohort, we conducted Kaplan–Meier analysis using PD-L1 TPS. We observed that patients with PD-L1 TPS ≥ 1% experienced worse survival outcomes, though not significant (*p* = 0.023; Fig. 5B). Notably, in line with methylation regulation mechanisms, a significant negative correlation emerged between cg19724470 methylation status and PD-L1 TPS, as determined by linear regression (*p* = 0.0025; Figure S4). However, the cg19724470 methylation status exhibited limited robustness in predicting the PD-L1 TPS in the PUMCH cohort (AUC = 0.630; Figure S5), indicating that complicated modification of PD-L1 expression exists besides promoter methylation in patients with PDAC.

To validate the prognostic significance of cg19724470 and PD-L1 TPS positivity, we conducted univariate and multivariate Cox regression analyses, considering various clinicopathological characteristics. Our results demonstrate that the methylation status of cg19724470 may independently predict OS in the PUMCH cohort, consistent with findings from the TCGA and ICGC cohorts (Fig. 5D; Table S9). Subsequently, we conducted the Kaplan–Meier survival analysis and found that the positivity of PD-L1 expression was not a significant prognostic factor in both the hypermethylated and hypomethylated subgroups of cg19724470 in the PUMCH cohort (*p* = 0.059; Fig. 5E, F).

### PD-L1 promoter methylation stratification by clinically crucial subgroups

**Fig. 6:**
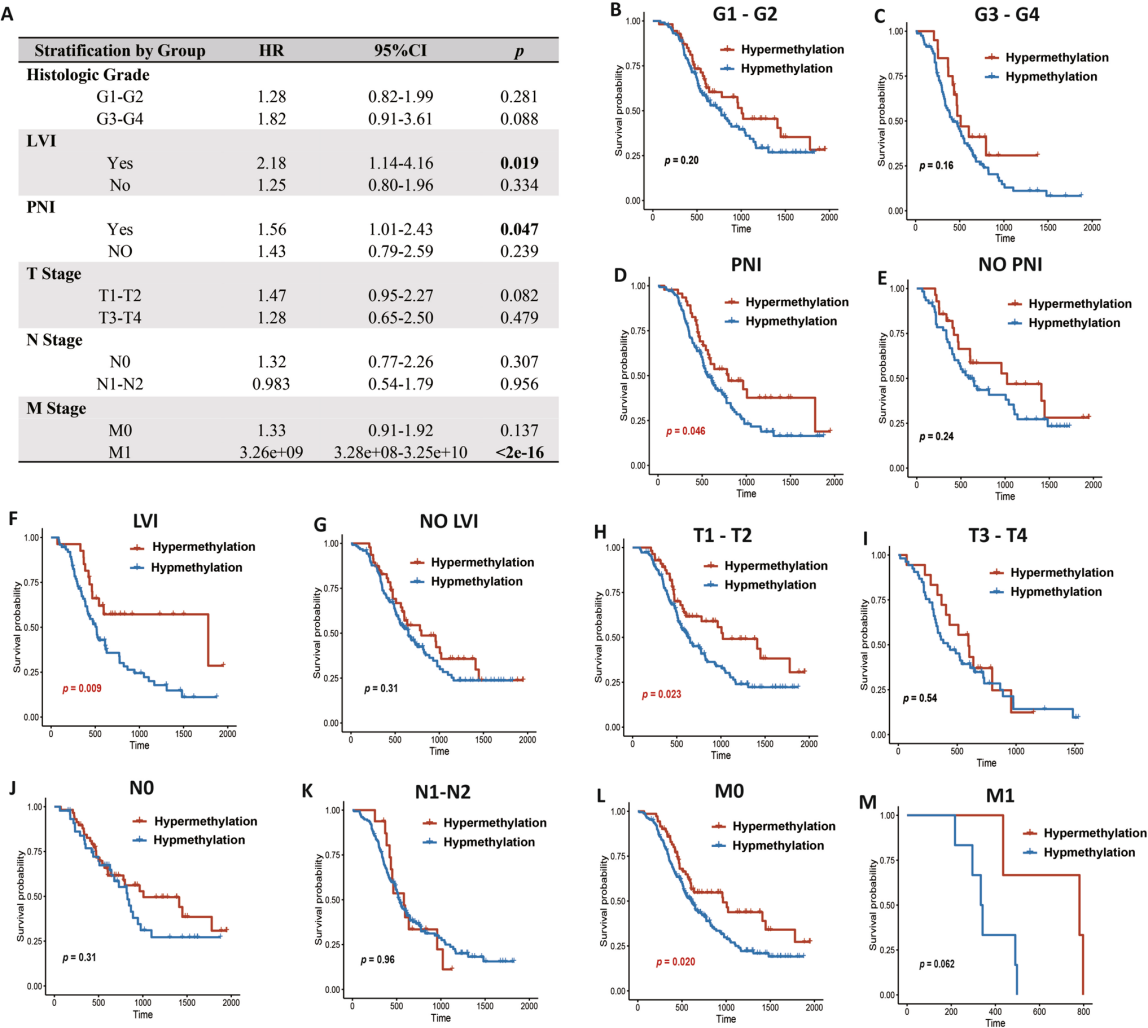
PD-L1 promoter methylation exhibited modification effect induced by PNI and LVI in the PUMCH cohort. **A**. Results of multivariate Cox analysis of PD-L1 promoter methylation per crucial clinical feature subgroups. *p* values < 0.05 were bolded; **B**–**K**. Kaplan–Meier curves of overall survival categorized by PD-L1 promoter methylation status in patients with histologic grade (**B**, **C**), PNI (**D**, **E**), LVI (**F**, **G**), T stage (**H**, **I**), N stage (**J**, **K**), and M stage (**L**, **M**). PNI, perineural invasion; LVI, lymphovascular invasion; HR, hazard ratio; CI, confidence interval. *p* values < 0.05 were marked in red

We conducted multivariate Cox analysis to assess the prognostic value of PD-L1 promoter methylation across various clinically significant subgroups, owing to its modifying influence on clinically crucial features. PD-L1 promoter methylation was categorized based on histological grade, perineural invasion (PNI), lymphovascular invasion (LVI), T stage, N stage and M stage status (Fig. 6A). Regarding OS, the findings indicated that PD-L1 promoter methylation had the most significant discriminative effect in patients with PNI (PNI: *p* = 0.047; No PNI: *p* = 0.239) and LVI (LVI: *p* = 0.019; No LVI: *p* = 0.334), and in patients with metastasis compared to those without metastasis (M0:* p* = 0.137; M1: *p* < 0.001). Subsequent Kaplan–Meier survival analysis for PD-L1 promoter methylation, stratified by these clinical features, confirmed the modifying effect induced by PNI, LVI, and distant metastasis (Fig. 6B–L).

## Discussion

Aberrant DNA methylation is an epigenetic phenomenon that significantly contributes to the dysregulation of gene expression during tumorigenesis and tumor progression. We conducted a comprehensive analysis of six methylation probes located in the PD-L1/PD-L2 genes, utilizing data from the TCGA, ICGC, and PUMCH cohorts. Our findings indicate that PD-L1 promoter methylation serves as a prominent prognostic marker and correlates with immune cell infiltration.

Consistent with the known association between hypermethylation of promoter regions and gene silencing, we observed a significant negative relevance between cg19724470 and PD-L1 expression. This correlation was validated using both IHC and pyrosequencing data from our PUMCH cohort, suggesting that alterations in cg19724470 methylation drive dysregulation of *CD274* expression in PDAC. Moreover, although not within gene promoter regions, we found a negative linear correlation between cg07211259 and *PDCD1LG2* expression, indicating that CpG sites outside the promoter regions of PD-L2 may also influence its expression regulation. Additionally, three probes (cg14133064, cg14374994, and cg14351952) in PD-L2 showed positive correlations with *PDCD1LG2* expression, implying distinct roles for CpG sites in different genomic regions and suggesting a complex regulatory mechanism in PD-L2.

Although negative correlations were observed in all three cohorts, the PUMCH cohort exhibited an undesirable predictive accuracy of PD-L1 expression based on PD-L1 promoter methylation status, indicating that cg19724470 methylation alone was insufficient as a biomarker for predicting PD-L1 expression. Besides altering methylation, many transcriptional regulators modulate PD-L1 expression in tumorigenesis and invasion [22]. It was reported that the alteration of histone methylation, such as histone H3 lysine 4 trimethylation (*H3K4me3*) and histone H3 lysine 27 trimethylation (*H3K27me3*), and acetylation, such as that mediated by histone deacetylase 3 (HDAC3), are involved in the regulation of PD-L1 expression [23–25]. Moreover, many transcriptional regulators exhibited the capacity to modulate PD-L1 expression. For instance, IRF1 and IRF2 could de-activate PD-L1 expression in hepatocellular carcinoma [26]. Thus, our results and those previous studies demonstrated that DNA promoter methylation is an important, but not the sole, factor that regulates PD-L1 expression, explaining the inconsistency between the PD-L1 promoter methylation status and PD-L1 expression in the PUMCH cohort.

Some mechanisms underlying the induction of PD-L1 promoter methylation were reported during tumor progression in solid tumors. For example, recruited TET2 binds to STAT1 and mediates the IFN-γ/JAK/STAT pathway, causing DNA demethylation of the PD-L1 promoter regions and thereby influencing the efficacy of immunotherapy [27]. Moreover, it was observed that global hypomethylation, not only the PD-L1 promoter methylation status, was positively associated with the expression of PD-L1 in melanoma, indicating that the expression of DNA methyltransferases could lead to PD-L1 expression and immunotherapy resistance [28]. Therefore, inhibitors of epigenetic regulators not only suppress tumor progression but also simultaneously increase PD-L1 expression; they could potentially be combined with anti-PD-L1 immunotherapy to improve therapeutic response.

To unveil the prognostic significance of PD-L1/PD-L2 methylation, we conducted both Cox and Kaplan–Meier analyses. Hypomethylation of cg19724470 was strongly associated with poor survival outcomes and was identified as an independent predictor of OS among the three PDAC cohorts. Notably, a unified conclusion regarding how PD-L1 methylation status influences survival outcomes has not been reached in pan-cancer analysis. Contrary to our conclusion, hypermethylation of PD-L1 has been identified to correlate with shorter OS in various tumors, including melanoma and colorectal cancer [15, 16]. However, high PD-L1 expression is associated with poor survival outcomes in many cancers, including renal, esophageal, breast, ovarian, and prostate cancers [29, 30], suggesting that hypomethylation of PD-L1 might lead to poor survival outcomes. These findings indicate that the methylation and expression status of PD-L1 play different biological roles in different cancers and have different impacts on patient survival. Therefore, using three large cohorts and two different methylation detection platforms, our results provide relatively stable and robust conclusions regarding how the PD-L1 promoter methylation status affects patient survival outcomes in PDAC. Moreover, it is worth noting that the methylation of cg19724470 was an independent prognostic factor in the PUMCH cohorts, while PD-L1 TPS was not independent. All these results indicated that PD-L1 promoter methylation could supplement PD-L1 TPS as a predictor of survival outcomes in clinical practice. Considering the clinicopathological characteristics, of our PUMCH cohort, we observed a discriminative effect of PD-L1 promoter methylation in patients with PNI, LVI and distant metastasis, indicating a better prognostic value of PD-L1 promoter methylation when combined with PNI, LVI, and distant metastasis.

Given the significance of PD-L1 in immunotherapy, we explored the association between PD-L1 promoter methylation status and immune cell infiltration, alongside established biomarkers and pathways relevant to immunotherapy. Our study revealed a significant negative relationship between CD4 + Th1 cells and PD-L1 promoter methylation across TCGA and ICGC cohorts. Despite numerous conflicting reports on the impact of CD4 + Th1 cells on the survival outcomes of PDAC patients, mechanistically, these cells are linked with key cytokines IL-2, IFN-γ, and TNF-α, contributing to anti-tumor immunity [31]. However, many studies have revealed that a high abundance of CD4 + Th1 cells is associated with poorer prognosis in various cancers, including PDAC, consistent with our findings indicating that hypomethylation of the PD-L1 promoter correlates positively with worse survival outcomes [32, 33]. One plausible explanation is the potential conversion of Th17 cells into Th1 cells in the presence of IL-12, resulting in distinct subsets of Th1 cells with varied biological functions [34]. Additionally, it is proposed that patients with increased infiltration of CD4 + Th1 cells may exhibit elevated PD-L1 expression [33], highlighting the potential role of epigenetic alterations in this association.

In general, PDAC is considered an immunologically "cold" tumor and exhibits a limited response to ICI immunotherapy. Various biological processes have been identified that contribute to the poor response to immunotherapy in patients with different PD-L1 promoter methylation statuses. Patients with hypermethylation tend to demonstrate enhanced resistance to immunotherapy through EMT, whereas hypomethylation is mediated through DNA damage repair processes, including homologous recombination and base excision repair. Different mechanisms have been proposed to enhance immunotherapy response within these subgroups. Patients with hypermethylation may benefit more from a combination of targeted EMT therapy, such as inhibitors blocking upstream signaling pathways including TGFβ, EGFR, and ICI immunotherapy [35]. The combination of ICI immunotherapy and DNA damage repair-based drugs, such as temozolomide, polyadenosine diphosphate-ribose polymerase inhibitors, and cisplatin, may be more effective in patients with hypomethylation [36]. The methylation status of the PD-L1 promoter is a potential biomarker associated with immunotherapy response and therapy selection.

Furthermore, although not as functional as cg19724470 in predicting survival outcomes, the other five probes, particularly cg02823866 and cg07211259, still displayed correlations with immune-related processes. Hu et al. identified 74 immune checkpoint genes that could predict responses to ICI immunotherapy and immune cell infiltration [17]. We correlated the expression/methylation profiles of PD-L1/PD-L2 with ICG expression using TCGA and ICGC cohorts. The results manifested that the expression/methylation profiles of PD-L1/PD-L2, particularly the methylation of cg02823866 and cg07211259, were significantly correlated with ICG expression. Specifically, the expression of PD-L1/PD-L2 displayed a significant positive relationship, whereas PD-L2 methylation status exhibited a significant negative relationship with the key immune checkpoint molecules *PD-1*, *CTLA4*, *LAG3*, and *TIGHT*. Although no significant association was observed between these molecules and PD-L1 promoter methylation, the methylation status of PD-L1/PD-L2 remains a potential biomarker for the effectiveness of bispecific antibody-based immunotherapy.

This study had some limitations. Methylation data from TCGA and ICGC were detected using the Infinium HumanMethylation450 BeadChip (Illumina 450K), which could not cover all CpG sites in PD-L1/PD-L2. Validation using our cohort only focused on cg19724470. Hence, other CpG sites could be identified as predictors of PDAC. With the development of methylation detection platforms, such as Infinium MethylationEPIC (Illumina 850K), and Infinium MethylationEPIC v2.0 BeadChip (Illumina 935K), a more accurate methylation map of PD-L1/PD-L2 in PDAC could be depicted. Another limitation of this study is the absence of a cohort that received ICI therapy to verify our conclusions. However, we primarily assessed the correlation between PD-L1 methylation, ICI response-related pathways, and immune cell infiltration in PDAC to elucidate their potential impact on the response to ICI immunotherapy. This requires further validation and exploration in future studies by establishing retrospective and prospective cohorts.

In summary, our study provides solid evidence that the methylation status of the PD-L1 promoter is a promising prognostic biomarker for PDAC, which may help predict patient clinicopathological features, survival outcomes, and distinguish immune characteristics. Moreover, PD-L1 promoter methylation exhibits the potential to predict the effectiveness of immunotherapy.

### Supplementary Information

Below is the link to the electronic supplementary material.

Supplementary file1 (PDF 2460 KB)

Supplementary file2 (PDF 153 KB)

## Data Availability

The datasets used and analyzed during the current study are available from the corresponding author on reasonable request.
