# Supplementary material for: Analysis of PD-L1 promoter methylation combined with immunogenic context in pancreatic ductal adenocarcinoma
Source: Cancer Immunol Immunother. 2024 Jun 4;73(8):149. doi: 10.1007/s00262-024-03745-y (PMC11150339; doi:10.1007/s00262-024-03745-y)
Supplement: Supplementary file 1 — Supplementary file1 (PDF 2460 KB) [file 262_2024_3745_MOESM1_ESM.pdf]

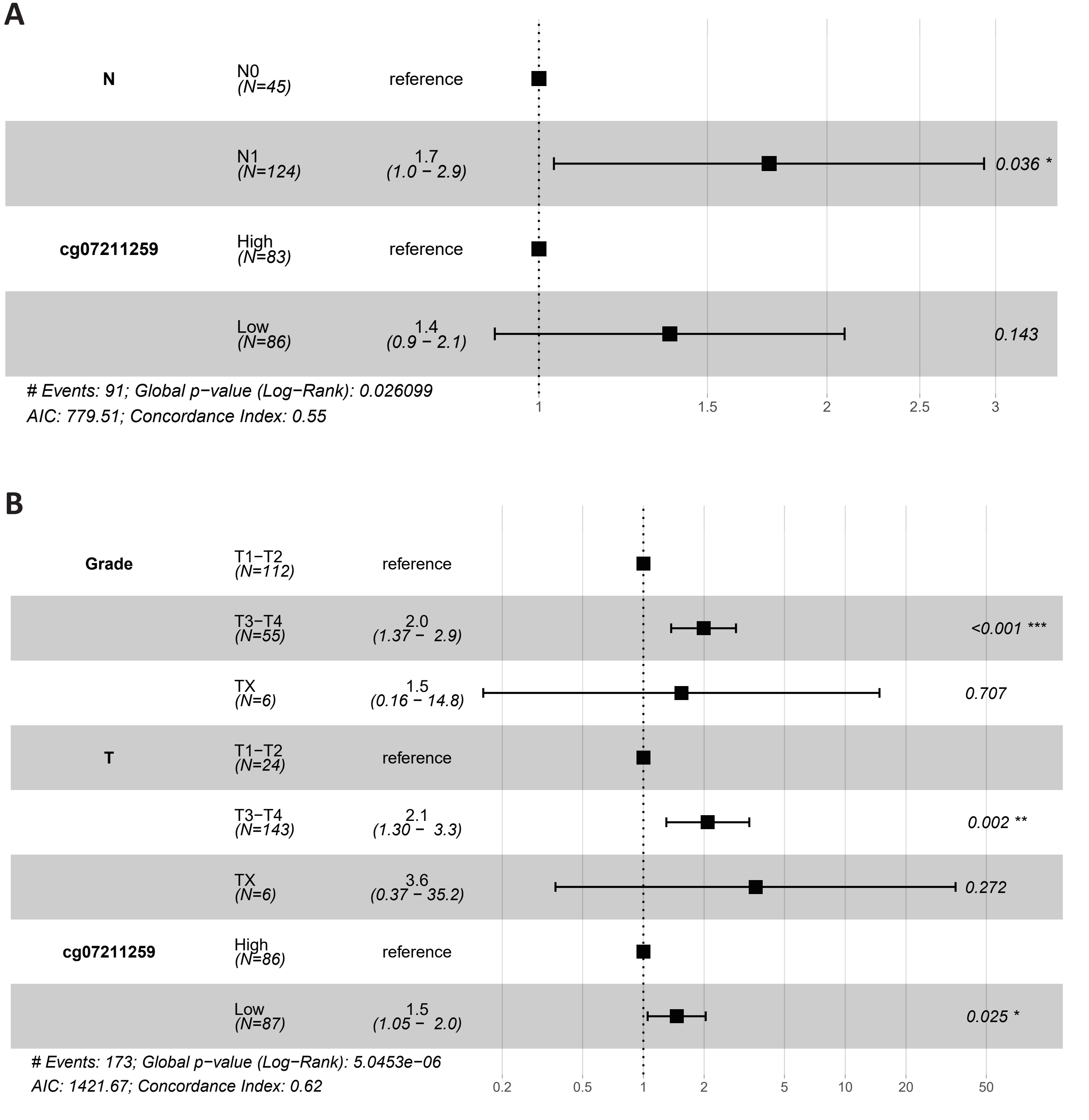


**Figure S1: Multivariate Cox analysis of cg07211259 methylation and the clinicopathological factors significant in the univariate Cox analysis ($p$ < 0.05) from the TCGA and ICGC cohorts, respectively**

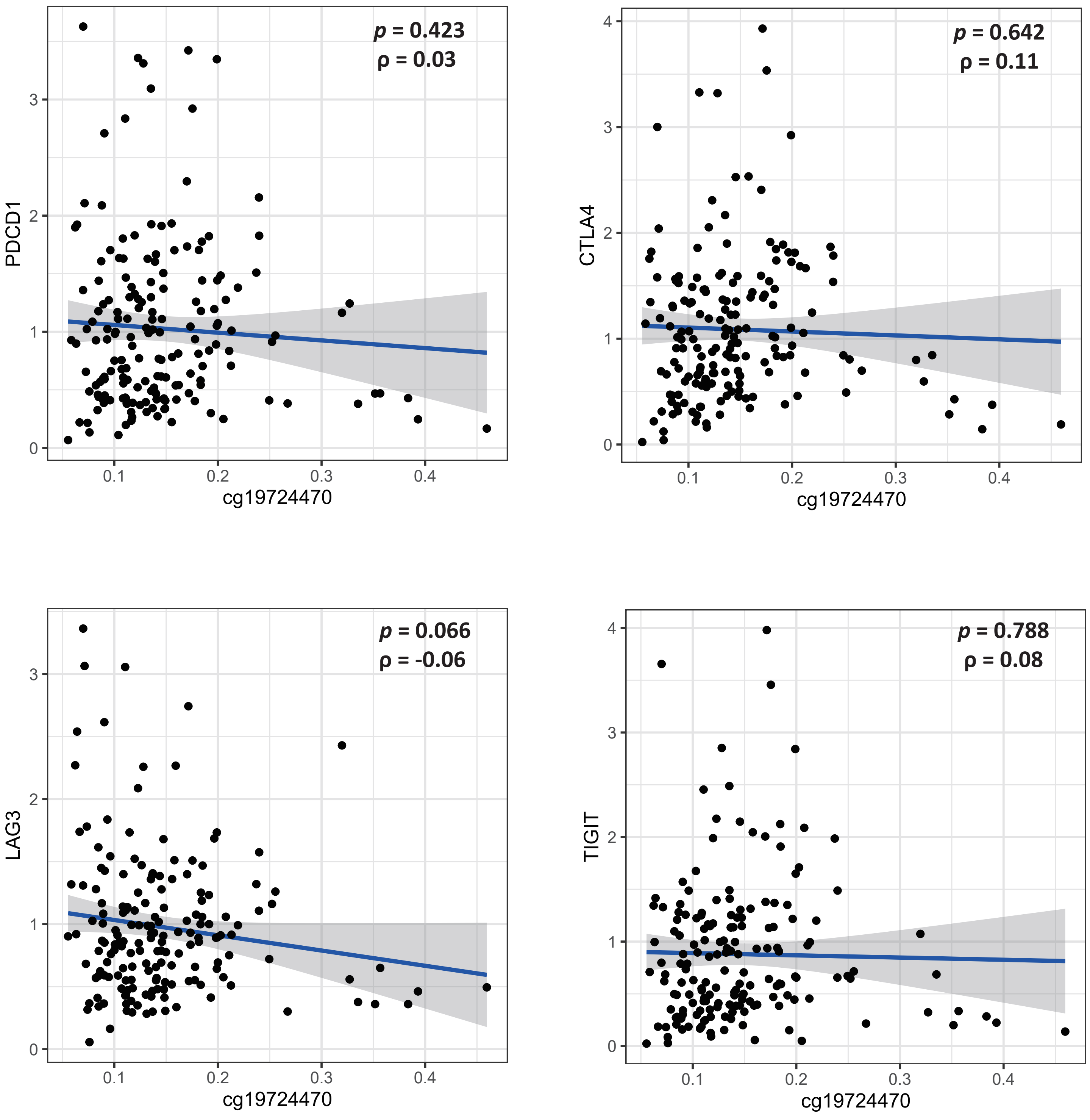


**Figure S2: The correlation of cg19724470 methylation with immune checkpoint molecules *PDCD1*, *CTLA4*, *LAG3*, and *TIGIT*, respectively, in the TCGA cohort**

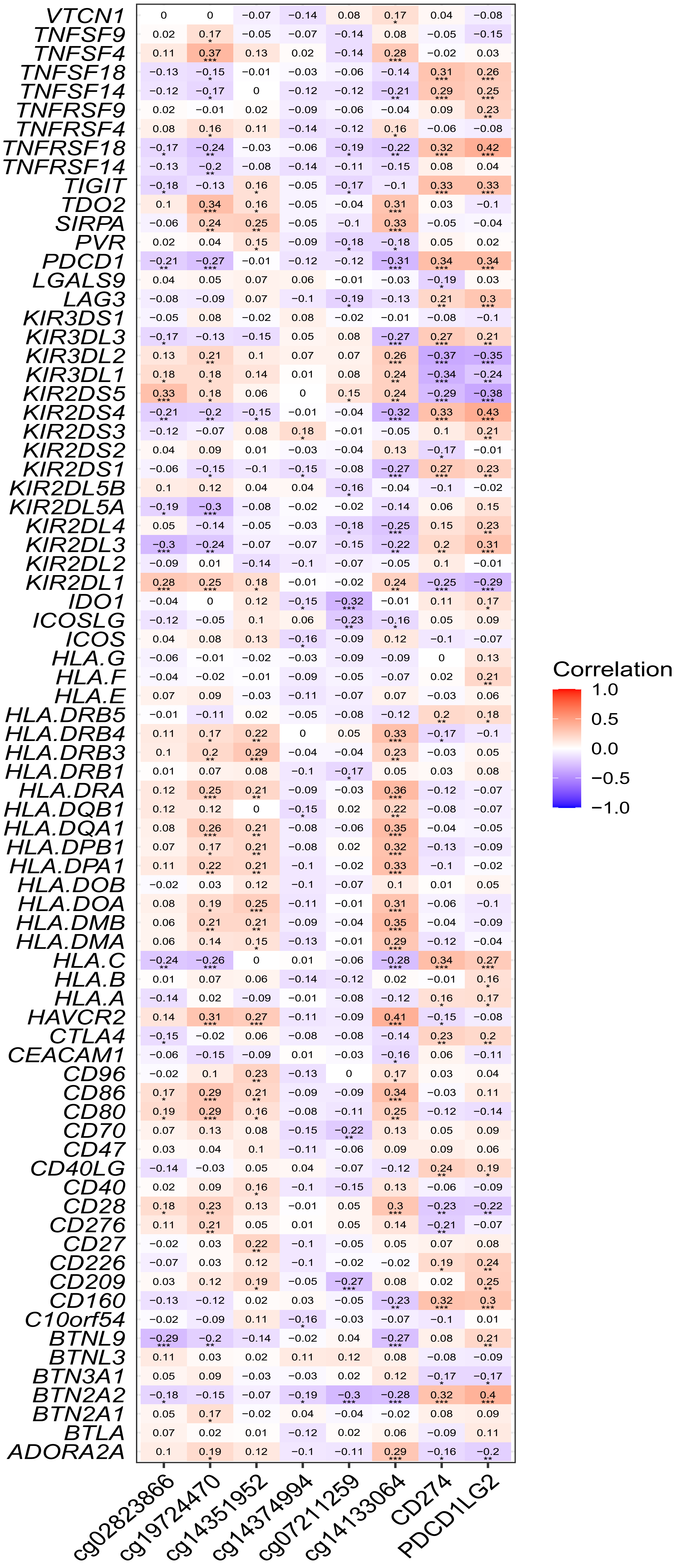


**Figure S3: The correlation heatmap of PD-L1/PD-L2 expression and related methylated CpG sites with 77 detected ICGs in the ICGC cohort**

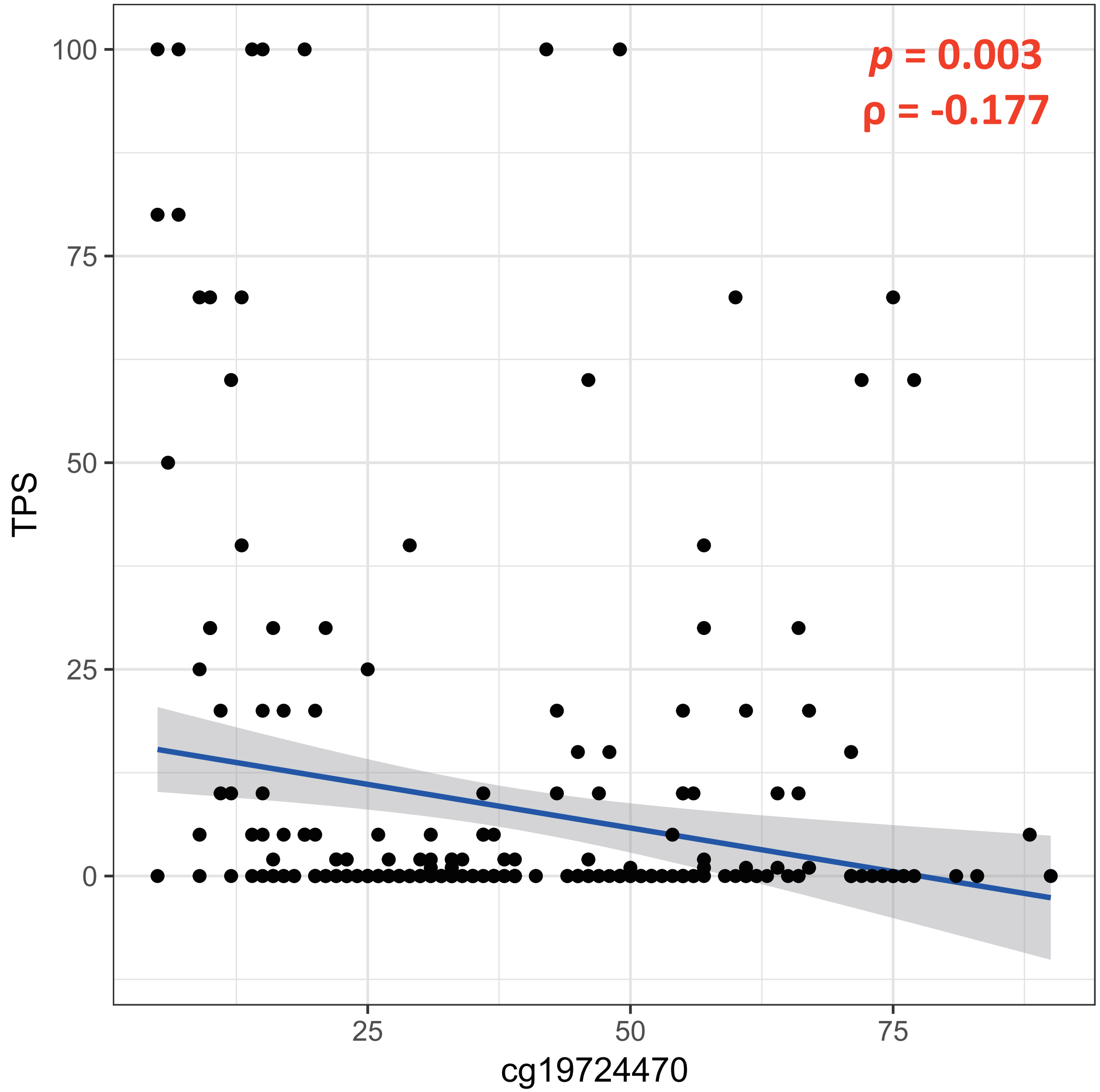


**Figure S4: The correlation of cg19724470 methylation status and PD-L1 TPS in the PUMCH cohort. TPS: tumor proportion score; *p* value < 0.05 was marked in red.**

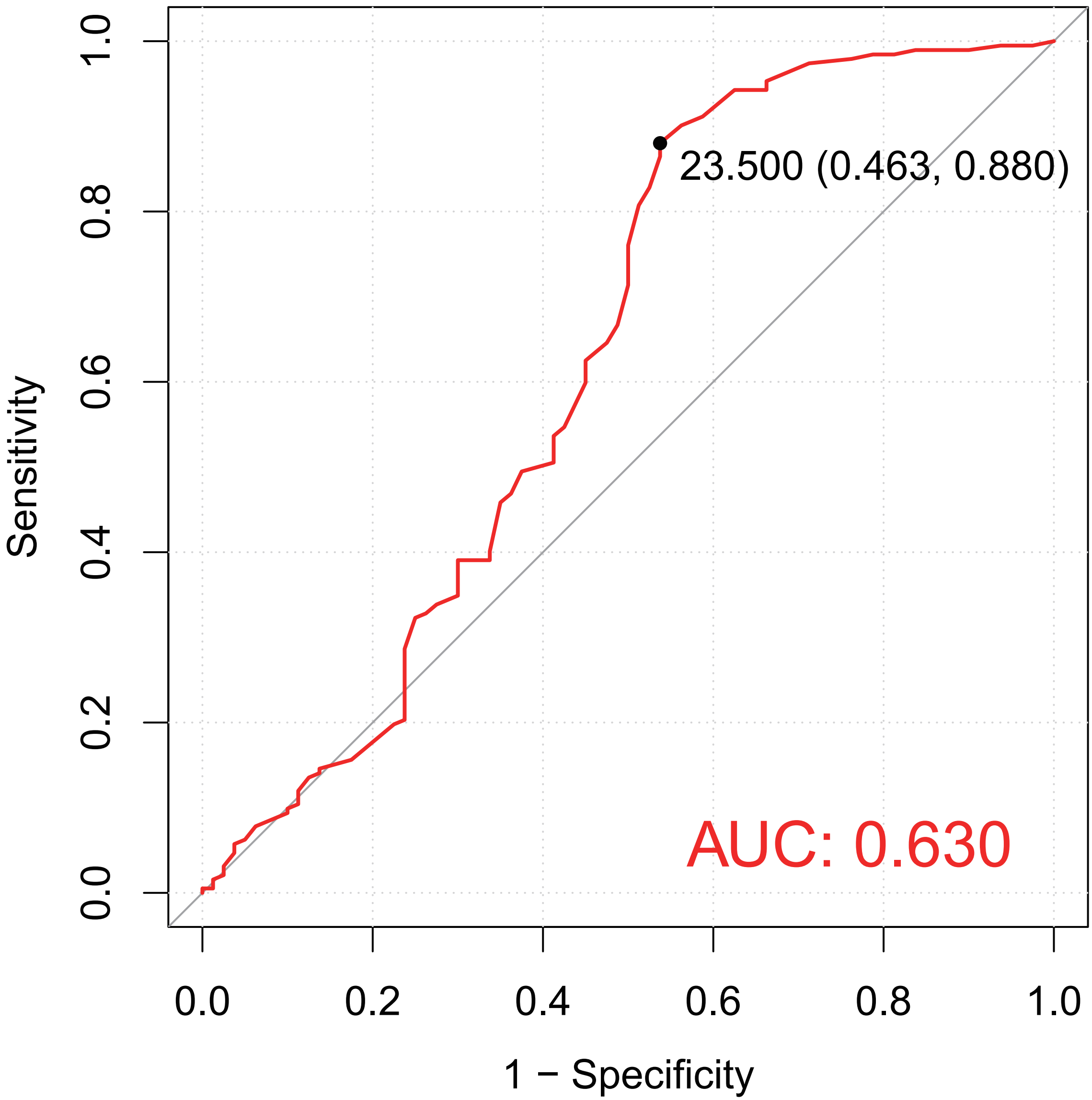


**Figure S5: The ROC curve for predicting PD-L1 TPS based on cg19724470 methylation status in the PUMCH cohort. AUC: area under curve.**
