# Supplementary material for: Analysis of PD-L1 promoter methylation combined with immunogenic context in pancreatic ductal adenocarcinoma
Source: Cancer Immunol Immunother. 2024 Jun 4;73(8):149. doi: 10.1007/s00262-024-03745-y (PMC11150339; doi:10.1007/s00262-024-03745-y)
Supplement: Supplementary file 2 — Supplementary file2 (PDF 153 KB) [file 262_2024_3745_MOESM2_ESM.pdf]

Table S1. The clinicopathological characteristics of the TCGA and ICGC cohorts

Variables	TCGA N(%)	ICGC N(%)
**Age**		
<60	53(30.64)	39(22.41)
≥60	120(69.36)	134(77.01)
Missing	0(0.00)	1(0.57)
**Sex**		
Male	95(54.91)	93(53.45)
Female	78(45.09)	81(46.55)
**Race**		
Asian	12(6.94)	NA
African American	6(3.47)	NA
White	151(87.28)	NA
Missing	4(2.31)	NA
**Tumor Location**		
Head	128(73.99)	NA
Body & Tail	27(15.61)	NA
Overlapping	18(10.40)	NA
**T Stage**		
T1-T2	25(14.45)	24(13.79)
T3-T4	147(84.97)	144(82.76)
Missing	1(0.58)	6(3.45)
**N Stage**		
N0	46(26.59)	45(25.86)
N1	124(71.68)	122(70.11)
NX	3(1.73)	7(4.02)
**M Stage**		
M0	80(46.24)	3(1.72)
M1	5(2.89)	8(4.60)
MX	88(50.87)	163(93.68)
**Histologic Grade**		
G1-G2	119(68.79)	113(64.94)
G3-G4	52(30.06)	55(31.61)
GX	2(1.16)	6(3.45)
**Maximum Tumor Dimension**		
<4cm	92(53.18)	NA
≥4cm	67(38.73)	NA
Missing	14(8.09)	NA

TCGA, The Cancer Genome Atlas; ICGC, International Cancer Genome Consortium; NA, not applicable.

Table S2. The clinicopathological characteristics of 291 PDAC patients in the PUMCH cohort

Variables	N(%)
**Age**	
<60	126(43.30)
≥60	165(56.70)
**Sex**	
Male	160(54.98)
Female	131(45.02)
**Histologic Grade**	
G1-G2	182(62.54)
G3-G4	109(37.46)
**Tumor Location**	
Head	178(61.17)
Body & Tail	113(38.83)
**PNI**	
No	96(32.99)
Yes	195(67.01)
**LVI**	
No	182(62.54)
Yes	109(37.46)
**T Stage**	
T1-T2	213(73.20)
T3-T4	78(26.80)
**M Stage**	
M0	281(96.56)
M1	10(3.44)

PNI, perineural invasion; LVI, lymphovascular invasion.

Table S3. Details of 6 probes located in PD-L1/PD-L2

Probe	chr	pos	strand	Address	Type	Islands_Name	Relation_to_Island	RefGene_Name	RefGene_Accession	RefGene_Group	Regulatory_Feature
**cg02823866**	chr9	5450410	-	6691157	II	chr9:5450409-5450629	Island	*CD274*	NM_014143	TSS200	Promoter Associated
**cg19724470**	chr9	5450936	+	86696312	II	chr9:5450409-5450629	S_Shore	*CD274*	NM_014143	5'UTR	Promoter Associated
**cg14351952**	chr9	5515324	+	97619110	II	NA	OpenSea	*PDCD1LG2*	NM_025239	5'UTR	NA
**cg14374994**	chr9	5543782	+	22666921	II	NA	OpenSea	*PDCD1LG2*	NM_025239	Body	NA
**cg07211259**	chr9	5510497	-	89663916	II	NA	OpenSea	*PDCD1LG2*	NM_025239	TSS200	Unclassified Cell type specific
**cg14133064**	chr9	5530115	+	30610977	II	NA	OpenSea	*PDCD1LG2*	NM_025239	Body	NA

NA, not applicable.

Table S4. Association of cg17924470, cg02823866, cg14351952, cg14374994, cg07211259, and cg14133064 methylation subgroups with clinicopathological characteristics in the ICGC cohort

Variables	N	PD-L1									PD-L2								
		cg02823866			cg19724470			cg14351952			cg14374994			cg07211259			cg14133064		
		Hyper	Hypo	*p*	Hyper	Hypo	*p*	Hyper	Hypo	*p*	Hyper	Hypo	*p*	Hyper	Hypo	*p*	Hyper	Hypo	*p*
**Age**				0.857			0.104			0.467			0.589			0.716			0.207
<60	39	19(48.72)	20(51.28)		24(61.54)	15(38.46)		22(56.41)	17(43.59)		21(53.85)	18(46.15)		18(46.15)	21(53.85)		16(41.03)	23(58.97)	
≥60	134	68(50.75)	66(49.25)		62(46.27)	72(53.73)		65(48.51)	69(51.49)		65(48.51)	69(51.49)		68(50.75)	66(49.25)		71(52.99)	63(47.01)	
Missing	1	0(0.00)	1(100.00)		1(100)	0(0.00)		0(0%)	1(100.00)		1(100.00)	0(0.00)		1(100.00)	0(0.00)		0(0.00)	1(100.00)	
**Sex**				0.181			0.034			0.181			0.381			0.381			0.064
Male	93	43(46.24)	50(53.76)		53(56.99)	40(43.01)		50(53.76)	43(46.24)		45(48.39)	48(51.61)		45(48.39)	48(51.61)		52(55.91)	41(44.09)	
Female	81	44(54.32)	37(45.68)		34(41.98)	47(58.02)		37(45.68)	44(54.32)		42(51.85)	39(48.15)		42(51.85)	39(48.15)		35(43.21)	46(56.79)	
**T Stage**				0.537			0.685			0.275			1			0.275			0.275
T1-T2	24	10(41.67)	14(58.33)		10(41.67)	14(58.33)		13(54.17)	11(45.83)		12(50.00)	12(50.00)		13(54.17)	11(45.83)		13(54.17)	11(45.83)	
T3-T4	144	73(50.69)	71(49.31)		74(51.39)	70(48.61)		73(50.69)	71(49.31)		72(50.00)	72(50.00)		73(50.69)	71(49.31)		73(50.69)	71(49.31)	
Missing	6	4(66.67)	2(33.33)		3(50.00)	3(50.00)		1(16.67)	5(83.33)		3(50.00)	3(50.00)		1(16.67)	5(83.33)		1(16.67)	5(83.33)	
**N Stage**				0.52			0.78			**0.041**			0.714			0.439			0.52
N0	45	21(46.67)	24(53.33)		24(53.33)	21(46.67)		28(62.22)	17(37.78)		25(55.56)	20(44.44)		21(46.67)	24(53.33)		24(53.33)	21(46.67)	
N1	122	61(50.00)	61(50.00)		59(48.36)	63(51.64)		58(47.54)	64(52.46)		59(48.36)	63(51.64)		64(52.46)	58(47.54)		61(50.00)	61(50.00)	
NX & Missing	7	5(71.43)	2(28.57)		4(57.14)	3(42.86)		1(14.29)	6(85.71)		3(42.86)	4(57.14)		2(28.57)	5(71.43)		2(28.57)	5(71.43)	
**M Stage**				0.789			0.455			0.455			0.62			0.169			0.294
M0	3	1(33.33)	2(66.67)		2(66.67)	1(33.33)		1(33.33)	2(66.67)		1(33.33)	2(66.67)		0(0.00)	3(100.00)		0(0.00)	3(100.00)	
M1	8	5(62.50)	3(37.50)		2(25.00)	6(75.00)		6(75.00)	2(25.00)		3(37.50)	5(62.50)		3(35.50)	5(62.50)		4(50.00)	4(50.00)	
MX & Missing	163	81(49.69)	82(50.31)		83(50.92)	80(49.08)		80(49.08)	83(50.92)		83(50.92)	80(49.08)		84(51.53)	79(48.47)		83(50.92)	80(49.08)	
**Histologic Grade**				1			0.508			0.308			0.167			**0.002**			0.284
G1-G2	113	56(49.56)	57(50.44)		53(46.90)	60(53.10)		58(51.33)	55(48.67)		58(51.33)	55(48.67)		67(59.29)	46(40.71)		57(50.44)	56(49.56)	
G3-G4	55	28(50.91)	27(49.09)		31(56.36)	24(43.64)		28(50.91)	27(49.09)		24(43.64)	31(56.36)		18(32.73)	37(67.27)		29(52.73)	26(47.27)	
GX & Missing	6	3(50.00)	3(50.00)		3(50.00)	3(50.00)		1(16.67)	5(83.33)		5(83.33)	1(16.17)		2(33.33)	4(66.67)		1(16.67)	5(83.33)	

PD-L1, programmed cell death ligand 1; PD-L2, programmed cell death ligand 2.

p values < 0.05 are bolded.

Table S5. Association of cg17924470, cg02823866, cg14351952, cg14374994, cg07211259, and cg14133064 methylation subgroups with clinicopathological characteristics in the TCGA cohort

Variables	N	PD-L1									PD-L2								
		cg02823866			cg19724470			cg14351952			cg14374994			cg07211259			cg14133064		
		Hyper	Hypo	*p*	Hyper	Hypo	*p*	Hyper	Hypo	*p*	Hyper	Hypo	*p*	Hyper	Hypo	*p*	Hyper	Hypo	*p*
**Age**				0.657			0.125			0.381			0.83			0.439			0.381
<60	53	25(47.17)	28(52.83)		31(59.49)	22(41.51)		29(54.72)	24(45.28)		27(50.94)	26(49.06)		24(45.28)	29(54.72)		29(54.72)	24(45.28)	
≥60	120	61(50.83)	59(49.17)		55(45.83)	65(54.17)		57(47.50)	63(52.50)		59(49.17)	61(50.83)		62(51.67)	58(48.33)		57(47.50)	63(52.50)	
**Sex**				**0.013**			0.759			0.11			0.2			0.396			0.813
Male	95	55(57.89)	40(42.11)		46(48.42)	49(51.58)		42(44.21)	53(55.79)		43(45.26)	52(54.74)		50(52.63)	45(47.37)		48(50.53)	47(49.47)	
Female	78	31(39.74)	47(60.26)		40(51.28)	38(48.72)		44(56.41)	34(43.59)		43(55.13)	35(44.87)		36(46.15)	42(53.85)		38(48.72)	40(51.28)	
**Race**				0.726			0.591			0.207			0.244			**0.044**			0.591
Asian	12	7(58.33)	5(41.67)		7(58.33)	5(41.67)		6(50.00)	6(50.00)		5(41.67)	7(58.33)		10(83.33)	2(16.67)		5(41.67)	7(58.33)	
African American	6	3(50.00)	3(50.00)		2(33.33)	4(66.67)		2(33.33)	4(66.67)		3(50.00)	3(50.00)		4(66.67)	2(33.33)		4(66.67)	2(33.33)	
White	151	73(48.34)	78(51.66)		74(49.01)	77(50.99)		74(49.01)	77(50.99)		74(49.01)	77(50.99)		71(47.02)	80(52.98)		74(49.01)	77(50.99)	
Missing	4	3(75.00)	1(25.00)		3(75.00)	1(25.00)		4(100.00)	0(0.00)		4(100.00)	0(0.00)		1(25.00)	3(75.00)		3(75.00)	1(25.00)	
**Tumor Location**				0.315			0.338			0.286			0.296			0.593			0.822
Head	128	60(46.88)	68(53.13)		61(47.66)	67(52.34)		60(46.88)	68(53.13)		62(48.44)	66(51.56)		65(50.78)	63(49.22)		62(48.44)	66(51.56)	
Body & Tail	27	17(62.96)	10(37.04)		13(48.15)	14(51.85)		14(51.85)	13(48.15)		12(44.44)	15(55.56)		11(40.74)	16(59.26)		14(51.85)	13(48.15)	
Overlapping	18	9(50.00)	9(50.00)		12(66.67)	6(33.33)		12(66.67)	6(33.33)		12(66.67)	6(33.33)		10(55.56)	8(44.44)		10(55.56)	8(44.44)	
**T Stage**				0.336			1			0.748			0.748			0.107			0.239
T1-T2	25	10(40.00)	15(60.00)		12(48.00)	13(52.00)		13(52.00)	12(48.00)		13(52.00)	12(48.00)		16(64.00)	9(36.00)		15(60.00)	10(40.00)	
T3-T4	147	76(51.70)	71(48.30)		73(49.66)	74(50.34)		72(48.98)	75(51.02)		72(48.98)	75(51.02)		69(46.94)	78(53.06)		70(47.62)	77(52.38)	
Missing	1	0(0.00)	1(100.00)		1(100.00)	0(0.00)		1(100.00)	0(0.00)		1(100.00)	0(0.00)		1(100.00)	0(0.00)		1(100.00)	0(0.00)	
**N Stage**				0.897			0.948			0.659			0.897			0.749			0.417
N0	46	22(47.83)	24(52.17)		23(50.00)	23(50.00)		21(45.65)	25(54.35)		22(47.83)	24(52.17)		24(52.17)	22(47.83)		19(41.30)	27(58.70)	
N1	124	62(50.00)	62(50.00)		61(49.19)	63(50.81)		64(51.61)	60(48.39)		62(50.00)	62(50.00)		60(48.39)	64(51.61)		65(52.42)	59(47.58)	
NX	3	2(66.67)	1(33.33)		2(66.67)	1(33.33)		1(33.33)	2(66.67)		2(66.67)	1(33.33)		2(66.67)	1(33.33)		2(66.67)	1(33.33)	
**M Stage**				0.676			0.546			0.396			0.885			0.885			0.741
M0	80	42(52.50)	38(47.50)		36(45.00)	44(55.00)		39(48.75)	41(51.25)		41(51.25)	39(48.75)		41(51.25)	39(48.75)		42(52.50)	38(47.50)	
M1	5	3(60.00)	2(40.00)		3(60.00)	2(40.00)		1(20.00)	4(80.00)		2(40.00)	3(60.00)		2(40.00)	3(60.00)		2(40.00)	3(60.00)	
MX	88	41(46.59)	47(53.41)		47(53.41)	41(46.59)		46(52.27)	42(47.73)		43(48.86)	45(51.14)		43(48.86)	45(51.14)		42(47.73)	46(52.27)	
**Histologic Grade**				0.491			0.271			0.808			0.702			0.555			0.374
G1-G2	119	61(51.26)	58(48.74)		61(51.26)	58(48.74)		61(51.26)	58(48.74)		62(52.10)	57(47.90)		59(49.58)	60(50.42)		58(48.74)	61(51.26)	
FG3-G4	52	25(48.08)	27(51.92)		23(44.23)	29(55.77)		24(46.15)	28(53.85)		23(44.23)	29(55.77)		25(48.08)	27(51.92)		28(53.85)	24(46.15)	
GX	2	0(0.00)	2(100.00)		2(100.00)	0(0.00)		1(50.00)	1(50.00)		1(50.00)	1(50.00)		2(100.00)	0(0.00)		0(0.00)	2(100.00)	
**Maximum Tumor Dimension**				0.675			0.098			0.373			0.778			0.267			0.972
<4cm	92	43(46.74)	49(53.26)		48(52.17)	44(47.83)		42(45.65)	50(54.35)		44(47.83)	48(52.17)		50(54.35)	42(45.65)		45(48.91)	47(51.09)	
≥4cm	67	35(52.24)	32(47.76)		33(49.25)	34(50.75)		35(52.24)	32(47.76)		34(50.75)	33(49.25)		28(41.79)	39(58.21)		34(50.75)	33(49.25)	
Missing	14	8(57.14)	6(42.86)		5(35.71)	9(64.29)		9(64.29)	5(35.71)		8(57.14)	6(42.86)		8(57.14)	6(42.86)		7(50.00)	7(50.00)	

PD-L1, programmed cell death ligand 1; PD-L2, programmed cell death ligand 2.

p values < 0.05 are bolded.

Table S6. Univariate Cox analysis of cg17924470, cg02823866, cg14351952, cg14374994, cg07211259, and cg14133064 from the TCGA and ICGC cohorts

Gene	methylation loci (low vs high expression)	TCGA		ICGC	
		HR(95%CI)	*p*	HR(95%CI)	*p*
**PD-L1**	**cg02823866**	0.933 (0.62-1.41)	0.743	1.23 (0.91-1.67)	0.175
	**cg19724470**	1.92 (1.25-2.95)	**0.003**	1.14 (0.84-1.54)	0.405
**PD-L2**	**cg14351952**	1.14 (0.58-1.33)	0.537	1.00 (0.74-1.35)	0.988
	**cg14374994**	1.10 (0.60-1.38)	0.657	0.95 (0.70-1.28)	0.716
	**cg07211259**	1.40 (0.92-2.13)	0.121	1.62 (1.19-2.19)	**0.002**
	**cg14133064**	0.99 (0.67-1.53)	0.944	1.19 (0.88-1.61)	0.254

PD-L1, programmed cell death ligand 1; PD-L2, programmed cell death ligand 2; TCGA, The Cancer Genome Atlas; ICGC, International Cancer Genome Consortium; HR, hazard ratio; CI, confidence interval.

p values < 0.05 are bolded.

Table S7. Univariate Cox analysis of clinicopathological characteristics from the TCGA and ICGC cohorts

Variables	TCGA		ICGC	
	HR(95%CI)	*p*	HR(95%CI)	*p*
**Age**				
<60	reference		reference	
≥60	1.32 (0.82 - 2.13)	0.246	0.96 (0.67 - 1.37)	0.820
**Sex**				
Female	reference		reference	
Male	0.75 (0.49 - 1.13)	0.172	1.22 (0.90 - 1.66)	0.196
**Grade**				
G1-G2	reference		reference	
G3-G4	1.31 (0.85 - 2.03)	0.213	1.98 (1.41 - 2.79)	**<0.001**
GX	0.89 (0.12 - 6.51)	0.915	2.75 (1.19 - 6.38)	**0.018**
**T Stage**				
T1-T2	reference		reference	
T3-T4	1.16 (0.61- 2.18)	0.657	1.62 (1.03 - 2.57)	**0.038**
TX	NA		3.64 (1.45 - 9.16)	**0.006**
**N Stage**				
N0	reference		reference	
N1	1.76 (1.05 - 2.96)	**0.031**	1.11 (0.79 - 1.57)	0.548
NX	NA		2.99 (1.33 - 6.73)	**0.008**
**M Stage**				
M0	reference		reference	
M1	0.72 (0.17 - 2.98)	0.648	0.68 (0.18 - 2.59)	0.577
MX	1.05 (0.69 - 1.60)	0.803	0.38 (0.12 - 1.19)	0.096
**Tumor Location**				
Head	reference		NA	
Body & tail	0.61 (0.31 - 1.20)	0.151	NA	
Overlapping	1.06 (0.57 - 1.96)	0.858	NA	
**Tumor Dimension**				
<4cm	reference		NA	
≥4cm	1.28 (0.84 - 1.95)	0.254	NA	
Missing	0.40 (0.12 - 1.30)	0.129	NA	

TCGA, The Cancer Genome Atlas; ICGC, International Cancer Genome Consortium; HR, hazard ratio; CI, confidence interval; NA, not applicable.

p values < 0.05 are bolded.

Table S8. Association of cg17924470 methylation and PD-L1 expression with clinicopathological characteristics in the PUMCH cohort

Variables	N	cg19724470			PD-L1 expression		
		Hyper	Hypo	*p*	Positive	Negative	*p*
**Age**				0.235			0.898
<60	126	30(23.81)	96(76.19)		37(29.37)	89(70.63)	
≥60	165	50(30.30)	115(69.70)		50(30.30)	115(69.70)	
**Sex**				0.895			0.608
Male	160	43(26.88)	117(73.13)		50(31.25)	110(68.75)	
Female	131	37(28.24)	94(71.76)		37(28.24)	94(71.76)	
**Histologic Grade**				**0.031**			0.186
G1-G2	182	58(31.87)	124(68.13)		49(26.92)	133(73.08)	
G3-G4	109	22(20.18)	87(79.82)		38(34.86)	71(65.14)	
**Tumor Location**				0.429			0.359
Head	178	46(25.84)	132(74.16)		57(32.02)	121(67.98)	
Body&Tail	113	34(30.09)	79(69.91)		30(26.55)	83(73.45)	
**PNI**				0.314			0.414
No	96	30(31.25)	66(68.75)		32(33.33)	64(66.67)	
Yes	195	50(25.64)	145(74.36)		55(28.21)	140(71.79)	
**LVI**				0.684			0.512
No	182	52(28.57)	130(71.43)		57(31.32)	125(68.68)	
Yes	109	28(25.69)	81(74.31)		30(27.52)	79(72.48)	
**T Stage**				1			0.885
T1-T2	213	59(27.70)	154(72.30)		63(29.58)	150(70.42)	
T3-T4	78	21(26.92)	57(73.08)		24(30.77)	54(69.23)	
**N Stage**				**<0.001**			**<0.001**
N0	103	58(56.31)	45(43.69)		17(16.50)	86(83.50)	
N1-N2	169	16(9.47)	153(90.53)		63(37.28)	106(62.72)	
**M Stage**				1			0.728
M0	281	77(27.40)	204(72.60)		85(30.25)	196(69.75)	
M1	10	3(30.00)	7(70.00)		2(20.00)	8(80.00)	

PNI, perineural invasion; LVI, lymphovascular invasion; PD-L1, programmed cell death ligand 1.
p values < 0.05 are bolded.

Table S9. Univariate Cox analysis of cg19724470 methylation, PD-L1 expression and clinicopathological characteristics in the PUMCH cohort

Variables	HR(95%CI)	*p*
**Age**		
<60	reference	
≥60	1.17 (0.87 - 1.59)	0.304
**Sex**		
Female	reference	
Male	1.34 (0.99 - 1.82)	0.0559
**LVI**		
No	reference	
Yes	1.21 (0.89 - 1.65)	0.214
**PNI**		
No	reference	
Yes	1.15 (0.83 - 1.58)	0.41
**AJCC**		
I-III	reference	
IV	2.44 (1.24 - 4.79)	**0.010**
**Tumor Location**		
Head	reference	
Body & tail	0.63 (0.46 - 0.87)	**0.004**
**cg19724470**		
hypermethylation	reference	
hypomethylation	1.53 (1.07-2.18)	**0.018**
**PD-L1 (TC)**		
≤1%	reference	
>1%	1.44 (1.05 - 1.96)	**0.024**

LVI: lymphovascular invasion; PNI: perineural invasion; AJCC, American Joint Committee on Cancer; PD-L1, programmed cell death ligand 1; TC, tumor cell; HR, hazard ratio; CI, confidence interval.

p values < 0.05 are bolded.
